# EPHA3 Contributes to Epigenetic Suppression of PTEN in Radioresistant Head and Neck Cancer

**DOI:** 10.3390/biom11040599

**Published:** 2021-04-18

**Authors:** Song-Hee Kim, Byung-Chul Kang, Daseul Seong, Won-Hyeok Lee, Jae-Hee An, Hyoung-Uk Je, Hee-Jeong Cha, Hyo-Won Chang, Sang-Yoon Kim, Seong-Who Kim, Myung-Woul Han

**Affiliations:** 1 Department of Otolaryngology, Ulsan University Hospital, University of Ulsan College of Medicine, Ulsan 44033, Korea; epoch55@naver.com (S.-H.K.); entkangbc@uuh.ulsan.kr (B.-C.K.); iop5075@naver.com (D.S.); say1004s@nate.com (W.-H.L.); wogml3546@naver.com (J.-H.A.); 2 Department of Radiation Oncology, Ulsan University Hospital, University of Ulsan College of Medicine, Ulsan 44033, Korea; jehu337@gmail.com; 3 Department of Pathology, Ulsan University Hospital, University of Ulsan College of Medicine, Ulsan 44033, Korea; heej0124@uuh.ulsan.kr; 4 Department of Otolaryngology, Asan Medical Center, University of Ulsan College of Medicine, Seoul 05505, Korea; chyowon@hotmail.com (H.-W.C.); sykim2@amc.seoul.kr (S.-Y.K.); 5 Department of Biochemistry and Molecular Biology, Asan Medical Center, University of Ulsan College of Medicine, Seoul 05505, Korea

**Keywords:** head and neck cancer, radioresistance, PTEN, EPHA3, C-myc, DNMT1, EZH2, DNA methylation, histone methylation

## Abstract

EPHA3, a member of the EPH family, is overexpressed in various cancers. We demonstrated previously that EPHA3 is associated with radiation resistance in head and neck cancer via the PTEN/Akt/EMT pathway; the inhibition of EPHA3 significantly enhances the efficacy of radiotherapy in vitro and in vivo. In this study, we investigated the mechanisms of PTEN regulation through EPHA3-related signaling. Increased DNA methyltransferase 1 (DNMT1) and enhancer of zeste homolog 2 (EZH2) levels, along with increased histone H3 lysine 27 trimethylation (H3K27me3) levels, correlated with decreased levels of PTEN in radioresistant head and neck cancer cells. Furthermore, PTEN is regulated in two ways: DNMT1-mediated DNA methylation, and EZH2-mediated histone methylation through EPHA3/C-myc signaling. Our results suggest that EPHA3 could display a novel regulatory mechanism for the epigenetic regulation of PTEN in radioresistant head and neck cancer cells.

## 1. Introduction

Radioresistance can lead to local recurrence and distant metastases in some patients with head and neck cancer treated with radiation. Activated PI3K/Akt/mTOR signaling is associated with radiotherapy and cytostatic drug resistance, probably through enhanced DNA repair [1,2]. The main negative regulator of this pathway is phosphatase and tensin homolog (PTEN). PTEN dephosphorylates phosphatidylinositol (3,4,5)-triphosphate (PIP3), a lipid second messenger produced following PI3K activation, to phosphatidylinositol (4,5)-bisphosphate (PIP2), thereby antagonizing the PI3K signaling pathway. PTEN also functions as a protein phosphatase in the cytoplasm, where it inhibits cell migration and induces cell cycle arrest, and in a phosphatase-independent manner in the nucleus to regulate chromosome stability, DNA repair, and apoptosis [3,4]. Loss-of-function mutations or epigenetic silencing of the PTEN tumor suppressor drives the development of a variety of human cancers, analogous to the direct oncogenic activation of PI3K proteins or its upstream activators [5].

An increasing number of studies have investigated the potential prognostic and predictive role of PTEN in cancer. However, the evaluation of gene mutations is not sufficient to fully uncover the broad range of activity loss status because of the complexity of PTEN regulation [6]. Genetic alterations and different mechanisms of regulation of PTEN expression and function, including transcriptional regulation, noncoding RNAs, post-translational modifications, and protein–protein interactions, have been reported [7,8]. PTEN function can be lost or inactivated by complete allelic losses, point mutations, or truncation mutations [7,8]. Epigenetic alterations also cause PTEN silencing through hypermethylation or mutations in the PTEN promoter region [8,9,10,11]. Aberrant methylation of this promoter is another mechanism for reduced PTEN expression. The hypermethylation of CpG islands in the PTEN promoter has been noted in various cancers [9,10,11,12,13,14]. CpG hypermethylation of the PTEN gene contributes to decreased expression of PTEN in acquired resistance to chemotherapeutic agents in breast and lung cancer cells [12,13].

EPHA3 is overexpressed in various cancers, such as glioblastoma, gastric cancer, and prostate cancer, and has an important role in tumorigenesis, aggressiveness, and radioresistance [15,16,17,18,19]. Some contradictory results underline the complexity of Eph receptor biology in cancer settings, and EPHA3 does not play a major role in lung and colorectal tumorigenesis [20,21]. However, we demonstrated previously that EPHA3 is overexpressed in radioresistant head and neck cancer, plays a crucial role in the development of radioresistance in head and neck cancers, and can regulate the epithelial mesenchymal transition pathway through the PTEN/Akt signal pathway [15]. Based on these previous findings, we focused on the epigenetic regulation of PTEN as related to radioresistance by EPHA3 in the present study. 

MYC is one of the most widely investigated oncogenes and transcription factors, being implicated in the formation, maintenance, and progression of different cancer types [22]. Regarding the relation of c-Myc and PTEN, it is known that c-Myc modulates genes associated with oncogenesis in glioblastoma through deregulation of miRNAs via the c-Myc-miR-26a-PTEN signaling pathway [23]. In the functioning of epigenetic alteration, Myc and DNMT3A form a ternary complex with Miz-1, and that this complex can corepress the p21Cip1 promoter [24] and Myc, and DNMT1 can impede the pleuripotent to totipotent state transition in embryonic stem cells [25]. Myc can interact with TRRAP, which recruits histone acetyltransferases, leading to a chromatin environment for transcription and tumor initiation [26]. In dual epigenetic regulation, EZH2 has been shown to interact with DNMT1 and dual DNMT1, and EZH2 mediated methylation silences some genes and promotes tumor progression [27,28,29]. Based on these findings, the evaluation of epigenetic modification in the translational control of PTEN through EPHA3 in radioresistant head and neck cancer revealed that EPHA3/C-myc-mediated DNMT1/EZH2 expression plays a critical role in the repression of PTEN transcription, necessary for Akt activation in radioresistance.

## 2. Materials and Methods

### 2.1. Cell Culture and Establishment of Radioresistant Head and Neck Cancer Cells and Reagents

Various head and neck cancer cell lines (AMC HN3, AMC HN3R, HN31, HN31R) and breast cancer cell lines (MDA-MB231, MDA-MB231R) were used in this study. AMC-HN3 cells were provided by Asan Medical Center (Seoul, Korea). HN31 cells were provided kindly by Dr. Jeffrey N. Myers, University of Texas, MD Anderson Cancer Center. MDA-MB231 cells were purchased from the American Type Culture Collection (ATCC, USA). Cancer cells were cultured in Dulbecco’s modified Eagle’s medium (DMEM; Invitrogen, Carlsbad, CA, USA) containing 10% fetal bovine serum (Invitrogen) and 100 μg/mL of penicillin/streptomycin and incubated at 37 °C with 5% CO_2_ in a humidified incubator. Head and neck cancer cells were grown to approximately 50% confluence in vented 75-cm^2^ culture flasks and irradiated with a 6-MV photon beam generated by a linear accelerator (CLINAC 600; Varian, Palo Alto, CA, USA) at a dose rate of 2 or 4 Gy. Cells were kept in continuous culture for <10 passages and tested using polymerase chain reaction (PCR) or fluorescence-activated cell sorting before evaluating the phenotype and expression of relevant proteins. Clinically relevant fractionated radiation doses (2 Gy) at 2-day intervals were delivered successively, using the previously established human laryngeal squamous cell carcinoma cells AMC HN3. An isogenic model of successively irradiated AMC HN3R cells was established after the cells received a cumulative dose of 70 Gy. This model was originally designed to investigate radioresistance, using cells of the same origin that differ only in terms of their radiosensitivity [15,30,31]. Previously, we performed a microarray expression data analysis in AMC HN3 and HN3R. We identified the EPHA3 overexpression in AMC HN3R and revealed that EPHA3 has an important role in radioresistance [15]. We prepared various additional radioresistant head and neck cancer cell lines (HN 31R cell line, MDA-MB-231R cell line) using the same procedure. 5-Aza-2′-deoxycytidine (A3656) and EZH2 inhibitors (SML0305) were purchased from Sigma-Aldrich (St. Louis, MO, USA). The c-Myc inhibitor (SC-213577) was purchased from Santa Cruz Biotechnology (Santa Cruz, CA, USA).

### 2.2. Transfection of EPHA3 CRISPR/dCas9 Activation Plasmids into Cultured HN3-AMC Cells

The CRISPR/dCas9 activation system was used for the overexpression of EPHA3 in AMC HN3 cells. The EPHA3 CRISPR/dCas9 activation plasmid (sc-401565-ACT; Santa Cruz Biotechnology, Dallas, TX, USA) consisted of a pool of three plasmids designed to overexpress the EPHA3 gene. The control CRISPR/dCas9 activation plasmid (sc-437275; Santa Cruz Biotechnology, Dallas, TX, USA) was used as a negative control. The plasmid transfection medium and UltraCruz transfection medium were used following the manufacturers’ protocol. Briefly, cells (1 × 10^5^ cells per well) were seeded on six-well culture plates in 3 mL antibiotic-free DMEM 24 h before transfection and grown to 70% confluence. Cells were transfected with 1 μg of the EphA3 CRISPR/dCas9 activation system using the UltraCruz transfection reagent (Santa Cruz Biotechnology, Dallas, TX, USA) and incubated at 37 °C with 5% CO_2_. Three days after transfection, cells were used for evaluation. EPHA3 expression was evaluated at 3 and 7 days, and at every experiment (supplementary Figure S1). 

### 2.3. RNA Interference

AMC HN3R cells were plated into six-well plates (1 × 10^5^ cells per well) 24 h before transfection. Cells were transfected with 30 nM of EPHA3 siRNA (sc-39934) or control siRNA (sc-37007). EphA3 shRNA and control construct was the mission lentivirus SCHLNV, Clone ID TRCN0000196830 (Sigma-Aldrich, Inc., St. Louis, MO, USA). The transfection reagent was from Santa Cruz Biotechnology. The cells were grown for 72 h before western blotting analysis. EPHA3 expression was evaluated at 3 and 7 days, and at every experiment (supplementary Figure S1). 

### 2.4. RNA Extraction and Quantitative Real-Time PCR Assay

Total RNA was isolated using a PureLink RNA Mini Kit (Ambion by Life Technologies™, Carlsbad, CA, USA), and cDNA was synthesized from 1 μg RNA with a Maxime RT-PCR PreMix Kit (iNtRON Biotechnology, Seoul, Korea). SYBR Green (Bio-rad, Hercules, CA, USA) PCR was performed in triplicate using a CFX96 Touch™ Real-Time PCR Detection System (Bio-rad, Hercules, CA, USA). All samples were normalized to the signal generated from β-actin. Data are presented as fold change (2−ΔΔCt) and were analyzed initially using Bio-rad CFX Software. All experiments were carried out more than three times. The level of mRNA expression is presented as the mean and standard error of three experiments.

### 2.5. Western Blotting Analysis

Total protein was extracted using PRO-PREP solution (iNtRON Biotechnology, Seoul, Korea). Protein concentrations were determined using a Pierce™ BCA Protein Assay Kit (Rockford, IL, USA). Equal amounts of protein were separated by SDS-PAGE and transferred to nitrocellulose membranes (Whatman, Maidstone, UK). Membranes were incubated with primary antibodies to EPHA3 (sc-920), DNMT1 (#5032), EZH2 (#5246), H3K27me3 (#9731), C-myc (#5605), phospho-Akt Ser473 (#4060), PTEN (#9551), and β-actin (sc-47778), all purchased from Cell Signaling Technology (Danvers, MA, USA). Membranes were then incubated with horseradish peroxidase (HRP)-conjugated secondary antibodies, followed by detection with a SuperSignal West Pico Trial kit (Thermo Fisher Scientific, Inc., Waltham, MA, USA) following the manufacturer’s instructions (Cayman Chemical, Ann Arbor, MI, USA). Western blotting analysis was performed at least three times, and representative figures are presented. The expression of the protein was quantified as compared to the beta-actin in the Western blot. 

### 2.6. Bisulfite Modification and Methylation Specific PCR (MSP) Assay

Genomic DNA (1 ug) was denatured with NaOH. Bisulfite treatment, during which methylated DNA is protected and unmethylated cytosine is converted to uracil, was carried out for 16 h at 50 °C on denatured genomic DNA. This procedure resulted in the conversion of unmethylated cytosine to thymine, but methylated cytosine remained unchanged. Then 1 ul of bisulfite modified DNA was amplified using primers that specifically amplify methylated or unmethylated DNA. PCR was performed in a final volume of 25 uL containing 10 mmol/L Tris Cl (pH 8.3), 50 mmol/L KCl, 1.25 mmol/L MgCl_2_, 100 umol/L dNTP 0.6 umol/L of each primer, 1 unit *Taq* DNA polymerase, and bisulfite-modified DNA (25 ng). Primer sequences of PTEN were as follows:

Unmethylated reaction, sense primer, 5′-GTGTTGGTGGAGGTAGTTGTTT-3′
Antisense primer, 5′-ACCACTTAACTCTAAACCACAACCA-3′
Methylated reaction, sense primer, 5′-TTCGTTCGTCGTCGTCGTATTT-3′
Antisense primer, 5′-GCCGCTTAACTCTAAACCGCAACCG-3′.

Qiagen reaction buffer and 1.5U HotStarTaq (Qiagen, Hilden, Germany) were used. PCR was performed as follows: One cycle of 95 °C for 5 min and 35 cycles of 94 °C for 45 s, 56 °C (Unmethylation) or 57.5 °C (Methylation) for 30 s, and 72 °C for 30 s, and then one cycle of 72 °C for 10 min. All amplified products were separated on 2% agarose gels, visualized using ethidium bromide, and photographed. 

### 2.7. Immunoprecipitation (IP)

Cells were resuspended in 50 mM Tris-HCL (pH 7.4), 1 mM EDTA, 1 mM EGTA, 150 mM NaCl, and 1% Triton X-100, supplemented with the protease and phosphatase inhibitor cocktail, and homogenized to prepare cell homogenates for IP. Cell homogenates (1 mL of homogenate containing 500 μg of protein) were precleared with 5 μg of normal rabbit IgG and 10 μg of protein A/G agarose (26146, Pierce Classic IP Kit, Thermo Fisher Scientific). The beads were washed with IP wash buffer (0.025 M Tris, 0.15 M NaCl, 0.001 M EDTA, 1% NP-40, 5% glycerol; pH 7.4). Mixtures were then incubated for 1 h at 4 °C and centrifuged at 12,000× *g* for 10 min at 4 °C. Samples were next incubated overnight at 4 °C with constant agitation with 5 μg of antibodies C-myc (sc-42) and normal rabbit IgG (sc-2027) as control. Protein A/G agarose beads were added later for another 1 h, and the immunoprecipitate was collected and washed thoroughly with wash buffer containing 0.5% Triton X-100. Each sample was then diluted with 1× SDS loading buffer, boiled for 10 min, and incubated with primary antibodies to c-Myc (sc-42), DNMT1 (#5032), EZH2 (#5246), and GAPDH (sc-47724) purchased from Cell Signaling Technology, and the immune complexes were precipitated by using mouse anti-rabbit IgG (light-chain specific) mAb (HRP conjugate) (A120-120P). Detection used a SuperSignal West Pico Trial kit (Thermo Fisher Scientific) following the manufacturer’s instructions (Cayman Chemical, Michigan, MI, USA).

### 2.8. Chromatin Immunoprecipitation (ChIP) Assays

ChIP assays used a Pierce™ Agarose ChIP Kit (Thermo Fisher Scientific, Waltham, MA, USA), following the manufacturer’s instructions. After the samples were harvested and washed twice with PBS, chromatin was cross-linked with 1% formaldehyde for 10 min at room temperature. Next, 1.25 M glycine was added to quench the excess formaldehyde. The cells were pelleted by centrifugation, resuspended in lysis buffer, and sonicated to generate 200 to 500 bp DNA fragments using a Bioruptor sonicator (Diagenode Inc., Sparta, NJ, USA). The diluted chromatin solution was immunoprecipitated with 2 μg of anti-EZH2 (Cell signaling, Danvers, MA, USA) or rabbit immunoglobulin G. After washing, cross-link reversal, DNA elution, and DNA purification, the relative amount of immunoprecipitated DNA was quantified via qPCR using the primers. Sequences of PCR primers are listed in the Additional File [32].

### 2.9. Immunohistochemical Analysis

For immunohistochemical evaluation, this study included 107 tissue specimens of 100 patients with laryngeal cancer. Regions of each primary tumor were chosen under microscopy and arranged pair-wise in tissue microarray blocks. The invasive front of each tumor was represented by two validated tissue cores on a tissue microarray. Fifty nine cases of primary total laryngectomy or laryngomicrosopic biopsy specimens and 45 cases of salvage total laryngectomy specimens for recurred cancer after radiotherapy or concurrent chemoradiotherapy were examined. After deparaffinization and rehydration, 4-μm thick sections were subjected to heat-induced antigen retrieval using a 0.01M citrate buffer (pH 6.0) for 1 h. Sections were incubated in aqueous 3% H_2_O_2_ for 15 min to quench endogenous peroxidase activity and then washed with 1× PBS. Slides were loaded into a humid chamber and blocked for 30 min with 1× universal blocking agent (10× Power Block™: BioGenex, San Ramon, CA, USA) before incubation overnight at 4 °C with primary antibodies against EphA3 (1:100; Sigma-Aldrich, Inc., St. Louis, MO, USA). The next day, slides were incubated for 1 h at room temperature and then treated with Envision Reagent (Dako REAL™ EnVision™, Glostrup, Denmark) for 30 min. Slides were washed with PBS and treated with the chromogen DAB for 15 min to allow formation of the brown reaction product. The slides were counterstained in Mayer’s hematoxylin, dehydrated in graded alcohol, cleared in xylene, and mounted. The slides were independently interpreted by two reviewers with no knowledge of the clinical data. Immunostaining was graded by the reactivity score (IRS), which reflects staining intensity (SI), assessed to be negative (=0), weak (=1), moderate (=2), or strong (=3). For further analysis, the specimens were divided into two groups: Negative with a score 0, and positive with a score 1–3 (supplementary Figure S2). 

## 3. Results

### 3.1. EPHA3 Maintains Sustained PTEN Suppression and Akt Activation through DNMT1-Mediated DNA Methylation in Radioresistant Head and Neck Cancer Cells

**Figure 1:**
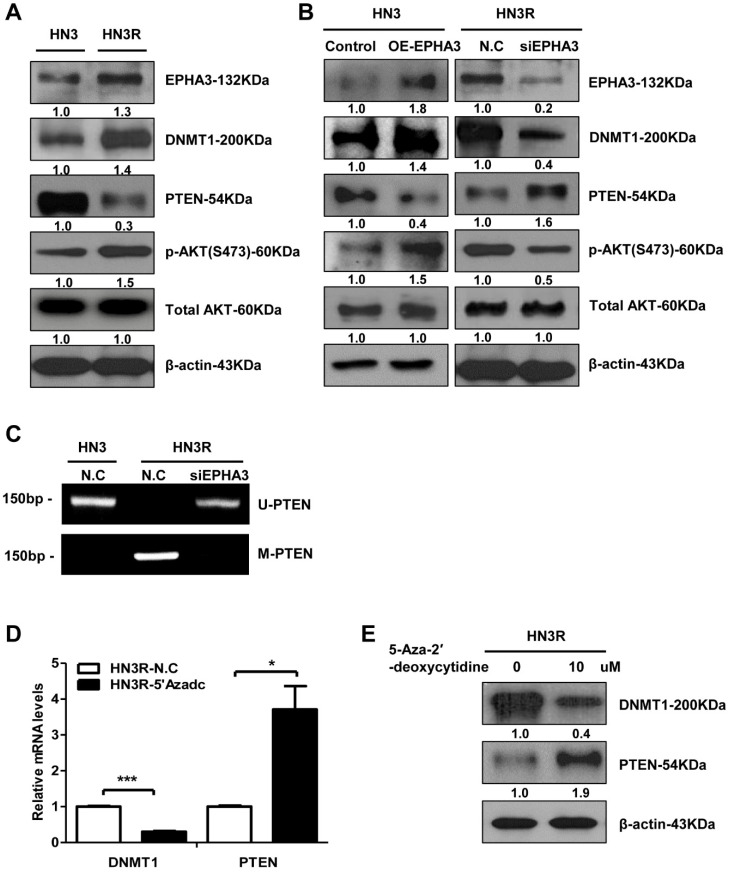
EPHA3 maintains PTEN suppression and Akt activation through DNMT-mediated DNA methylation in radioresistant head and neck cancer cells. (**A**) Western blotting analysis showing the expression levels of EPHA3, DNMT1(DNA methyltransferase), PTEN, pAkt, and total Akt in HN3 and HN3R cells. (**B**) Expression levels of EPHA3, DNMT1, PTEN, pAkt, and total Akt were assessed using western blotting in HN3 cells after transfection with EPHA3, and expression levels of EPHA3, DNMT1, PTEN, pAkt, and total Akt were assessed using western blotting in HN3R cells after the silencing of EPHA3 (**C**). A conventional method of testing the methylation status, the MSP assay, showed aberrant, hypermethylated DNA for PTEN in HN3 and HN3R cells. (**D**,**E**) Expression of PTEN at mRNA and protein levels after treatment with the DNA demethylating agent 5-aza-20-deoxycytidine (5-AZAdC) in HN3R cells using RT-PCR and western blotting. * *p* < 0.05; *** *p* < 0.001.

At first, we investigated the epigenetic regulation of PTEN by EPHA3 in radioresistant head and neck cancer cells. DNMT1 (DNA methyltransferase) levels with EPHA3 were increased significantly in HN3R cells compared to parent HN3 cells (Figure 1A). The activation of Akt is a key mediator of radiation resistance, and we evaluated levels of Akt and PTEN for negative regulation by the PI3K/Akt pathway. PTEN loss and activation of Akt was observed in HN3R cells compared to HN3 cells (Figure 1A). We evaluated the expression of proteins after the transfection of EPHA3 in HN3 cells to explore the effects of the EPHA3 expression level on DNMT1 and Akt and on the suppression of PTEN (Figure 1B). EphA3 overexpression upregulated DNMT1 and phospho-Akt expression and downregulated PTEN. Furthermore, we identified the effect of EPHA3 silencing in HN3R cells (Figure 1B). Western blotting showed that the inhibition of EPHA3 with siRNA downregulated DNMT1 and Akt activation and caused increased PTEN expression. 

For confirmation of DNA methylation effect on PTEN expression through EPHA3, we performed an MSP assay, a conventional method to test the methylation status, and the result showed that the HN3 cells displayed normal unmethylation of the PTEN promoter, but the HN3R cells exhibited aberrant, hypermethylated DNA for this promotor. After the silencing of EPHA3 in HN3R, the unmethylated PTEN level was increased (Figure 1C). To confirm the epigenetic regulation of PTEN by DNMT1 in HN3R cells, HN3R cells were treated on 2 successive days with 10 µM of 5-AZAdC (DNA demethylating agent) and we examined the expression of mRNA and protein levels of PTEN in HN3R cells. DNMT1 mRNA and protein in whole-cell lysates were reduced, and PTEN expression was increased markedly at both mRNA and protein levels (Figure 1D,E). These results suggest that EPHA3 maintains a sustained PTEN suppression through DNMT-mediated-DNA methylation.

### 3.2. EPHA3 Maintains PTEN Suppression and Akt Activation through EZH2-Mediated Histone Methylation in Radioresistant Head and Neck Cancer Cells

**Figure 2:**
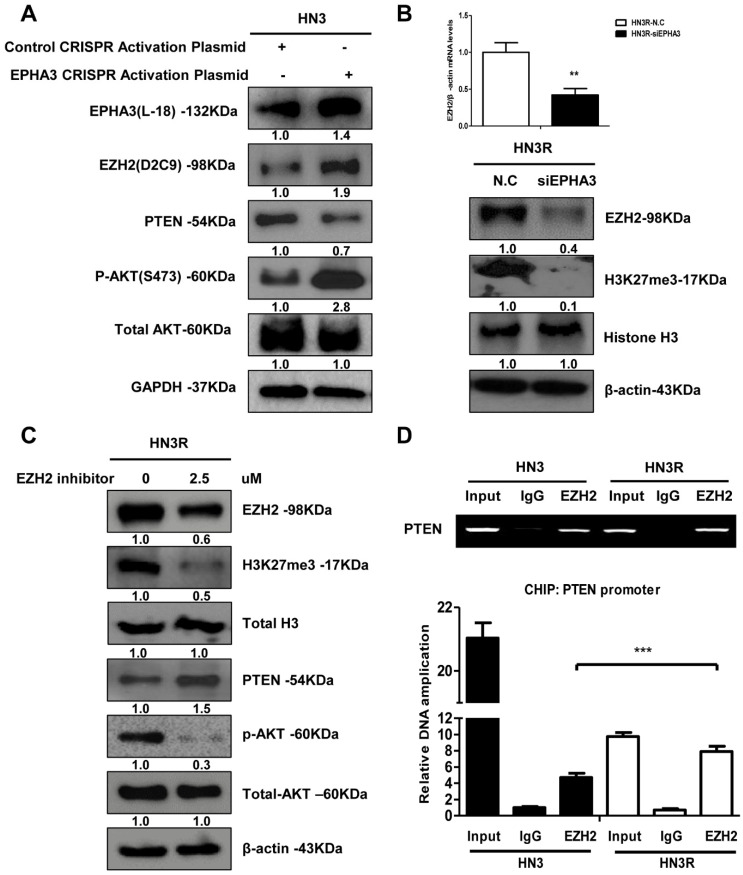
EPHA3 maintains PTEN suppression and Akt activation through EZH2-mediated histone methylation in radioresistant head and neck cancer cells. (**A**) Protein levels of EZH2, PTEN, pAkt, and total Akt in EPHA3 transfected HN3 cells. (-: control, +; CRISPR Activation Plasmid transfection) (**B**) Protein levels of EZH2, H3K27me, and total H3 after the silencing of EPHA3 in HN3R cells, and in EPHA3-overexpressing HN3 cells. ** *p* < 0.01 (**C**) Expression of PTEN, total H3, pAKT, and total Akt through suppression of H3K27me3 after treatment with the EZH2 inhibitor (2.5 µM). (**D**) ChIP assays showing endogenous EZH2 bound to the promoter region of PTEN. *** *p* < 0.001.

The enhancer of zeste homolog 2 (EZH2) is a methyltransferase and the core catalytic subunit of polycomb repressive complex 2, and this enzyme is essential for the epigenetic maintenance of lysine 27 residues of the histone H3 trimethylation (H3K27me3) repressive chromatin marker [9]. EZH2 mediates modifications in histone methylation, resulting in the downregulation of numerous tumor suppressor genes, including PTEN [33]. EZH2 binds directly to the PTEN promoter to regulate transcription [32] in gastric cancer. Based on these findings, EZH2, histone H3-lysine 27 methyltransferase (H3K27me3), and PTEN expression was evaluated in HN3 and HN3R cells. At first, we assessed EZH2 and H3K27me3 in EPHA3 overexpressing HN3 to determine if EPHA3 modulates EZH2 expression. After transfection of EPHA3 in HN3 cells, EZH2 and phosphor-Akt expression was increased with suppression of PTEN (Figure 2A). EZH2 expression increased with H3K23me3 and decreased after the silencing of EPHA3 in HN3R cells (Figure 2B). In addition, we investigated whether treatment with the EZH2 inhibitor (2.5 µM) could modulate PTEN expression epigenetically through H3K27me3. EZH2 inhibition induced the reactivation of PTEN through H3K27me3 suppression (Figure 2C). Additionally, we proposed that EZH2 binds to the PTEN promoter region directly, and used ChIP assays to test this hypothesis. We divided the PTEN promoter into ten sequences to determinate the exact binding region. We identified that EZH2 was conjoined directly to the PTEN promoter from 754 to 954 bp (Figure 2D), consistent with the transcriptional control of PTEN expression. We confirmed these results in another head and neck cancer cell line (HN31) and breast cancer cell line (MDA-MB-231) using western blot. We found that PTEN was suppressed with elevated EPHA3, DNMT1, and EZH2 expression in other radioresistant cell lines (Supplementary Figure S3). Thus, EPHA3 maintains PTEN suppression through EZH2-mediated histone methylation with DNMT1-mediated DNA methylation in radioresistant head and neck cancer cells.

### 3.3. C-Myc Could Mediate the Functional Link between EPHA3 and DNMT1/EZH2 That Regulates PTEN-Based Radiation Resistance Epigenetically

**Figure 3:**
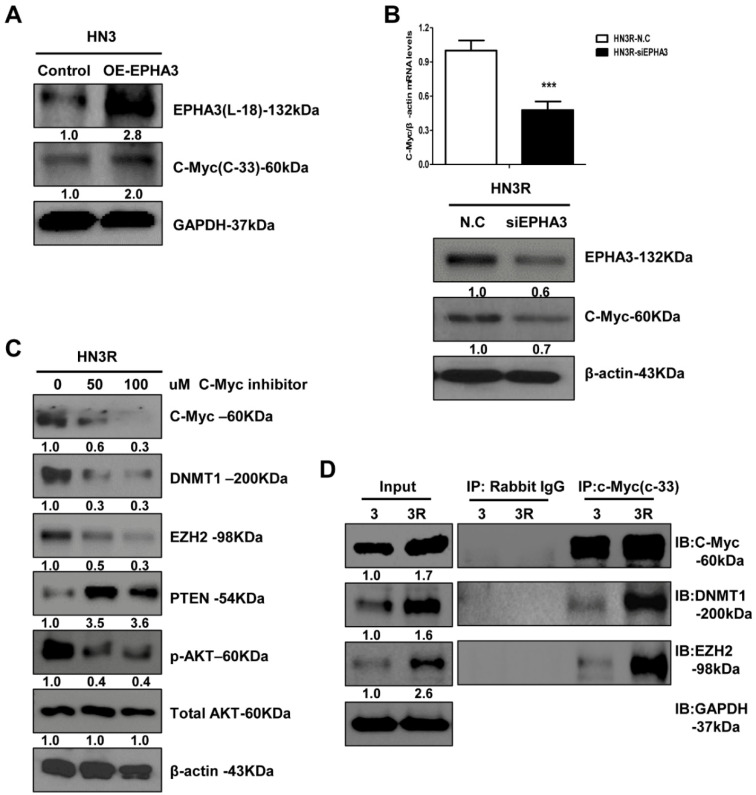
PTEN is repressed by EZH2-mediated histone methylation and DNMT-mediated DNA methylation via the EPHA3/c-Myc pathway in radioresistant head and neck cancer cells. (**A**) C-Myc protein expression after transfection of EPHA3 in HN3 cells. (**B**) C-Myc mRNA and protein expression after the silencing of EPHA3 in HN3R cells. (**C**) Expression of EZH2, DNMT1, PTEN, pAkt, and total Akt after treatment with the c-Myc inhibitor. (**D**) Assessment of direct interaction between DNMT1/EZH2 and c-myc using immunoprecipitation (IP). *** *p* < 0.001.

We investigated the role of c-Myc as a linking molecule between EPHA3 and EZH2 or DMNT1. Initially, we explored the EPHA3 regulation of c-Myc expression. We found that c-Myc protein expression was upregulated after the transfection of EPHA3 into HN3 cells (Figure 3A), and c-Myc mRNA and protein expression were downregulated by the silencing of EPHA3 in HN3R cells (Figure 3B). Furthermore, the inhibition of c-Myc reduced the expression of EZH2 and DNMT1 and upregulated the expression of PTEN with decreased Akt activation (Figure 3C). We also used IP to confirm the direct regulation of DNMT1 and EZH2 through c-Myc. DNMT1 and EZH2 were highly expressed in AMC HN3R cells (Figure 3D), consistent with the increase in c-Myc protein levels. EPHA3-induced c-Myc expression regulates DNMT1 and EZH2 at the transcriptional level in radioresistant head and neck cancer cells. 

### 3.4. EPHA3 Inhibition Suppresses Tumor Growth through c-Myc, DNMT1, EZH2, and PTEN in an AMC HN3R Xenografts Model

**Figure 4:**
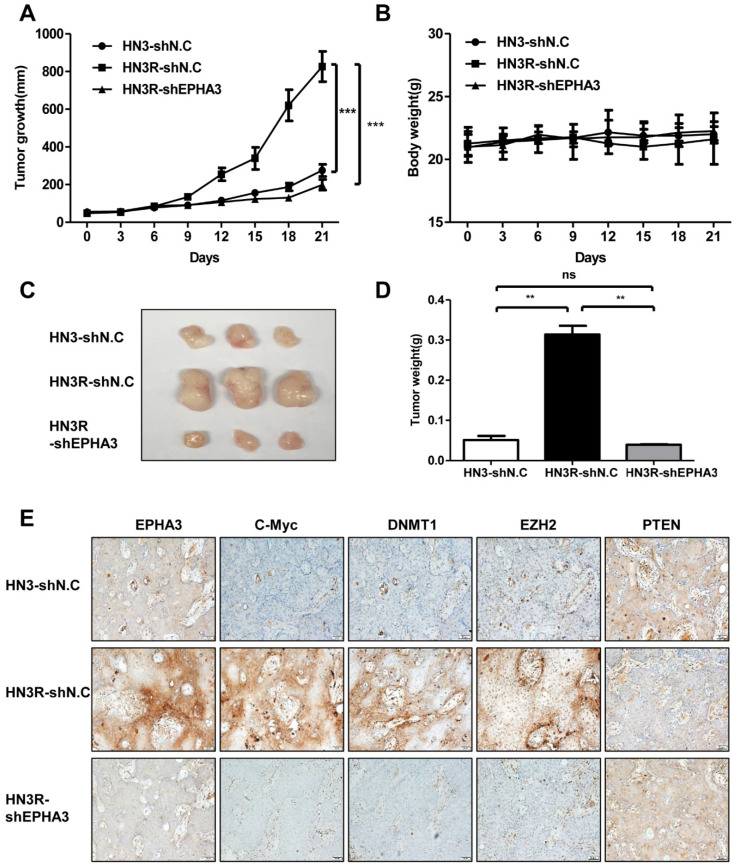
EphA3 inhibition suppress tumor growth through regulation of c-myc, DNMT1/EZH2, and PTEN in an AMC HN3R xenografts model. **(A**)Tumors were measured regularly, and the relative tumor volume was tracked for each animal (each group: 4 mice). (**B**) Body weight was measured every three days. (**C**) The photographs show representative tumors from each mouse of approximate 3 weeks. (**D**) Tumor weight was examined in each treatment groups at 21 days. (**E**) Immunohistochemical staining was performed in mouse tumor specimens. Representative photographs are shown. ** *p* < 0.005; *** *p* < 0.0001.

Previously, we confirmed the biological efficacy of EPHA3, using AMC HN3R in vitro and in vivo, related to radioresistance [15]. Having established the in vitro effects of the silencing of PTEN through EPHA3/C-myc and DNMT1/EZH2 in radioresistant cancer cells, the biologic efficacy of EPHA3 was tested in tumor growth delay, and expression of the proteins was evaluated using AMC HN3R xenografts in nude mice. Tumor mice (each group with 4 mice) were injected with AMC HN3 cell line vehicle, AMC HN3R cell line, and AMC HN3R cell line with EPHA3 shRNA treatment. EPHA3 inhibition resulted in a noticeable tumor growth delay at 21 days, compared with the AMC HN3R group (Figure 4A) Mouse body weight monitoring suggested that all treatments were relatively well tolerated (Figure 4B). In Figure 4C, representative tumors from each mouse at 21 days was shown. EPHA3 inhibition showed significantly decreased tumor weight compared with mice in control group (AMC HN3R group) at 21 days (Figure 4D). Furthermore, the expression of EPHA3, c-Myc, DNMT1, EZH2, and PTEN was assessed with IHC using tumor specimens from mice. We identified the overexpression of EPHA3, c-Myc, DNMT1, and EZH2 with decreased expression of PTEN in the AMC HN3R group compared with AMC HN3 group on IHC. After silencing of EPHA3 in AMC HN3R, the expression of EPHA3, c-Myc, DNMT1, and EZH2 decreased and PTEN was overexpressed. (Figure 4E). These data suggest that EPHA3 can induce PTEN suppression in radioresistant head and neck cancer. 

### 3.5. EphA3 and c-Myc Is Overexpressed and PTEN Expression Is Decreased in Recurrent Laryngeal Cancer Specimens

For immunohistochemical evaluation, this study included 104 tissue specimens that underwent partial or total laryngectomy with laryngeal cancer. In 45 patients, salvage surgery was performed after radiation (N = 35) and concurrent chemoradiation (cisplatin, N = 10). Most of the patients received conventional radiotherapy, and the mean radiation dose was 67.5 Gy (64.5-70 Gy). In 59 patients, primary surgery was done as an initial treatment. (supplementary Table S1). In our previous study, the staining score for EphA3 expression in 45 salvage surgical specimens after radiation failure was significantly higher than that in 59 surgical specimens without radiation treatment, according to an univariate analysis (*p* = 0.016) [15]. We additionally performed IHC of c-Myc and PTEN. EPHA3 was expressed weakly in 32 tumors, moderately in 24 tumors, and strongly in 9 tumors. C-myc was expressed weakly in 19 tumors, moderately in 27 tumors, and strongly in 5 tumors. PTEN was expressed weakly in 21 tumors, moderately in 25 tumors, and strongly in 5 tumors. We found overexpression of c-Myc and decreased expression in PTEN in recurrent laryngeal cancer specimens (Table 1). Collectively, these results suggest that EphA3 and c-Myc are overexpressed, and PTEN is suppressed, in radioresistant head and neck cancer, and that may this play a crucial role in the development radioresistance in head and neck cancers. 

## 4. Discussion

EPHA3 expression is associated with tumor promotion in some cancer types, while it has tumor suppressor roles in others [17,20,21,34]. However, previously, we found that EPHA3 can maintain radioresistance in head and neck cancer through epithelial mesenchymal transition [15]. PTEN can regulate cancer stem cell development and maintenance, specifically affecting critical features of these cells through downstream signaling pathways, such as the PI3K/Akt pathway [3,5]. PTEN loss can influence tumor progression, metastasis, and radiation and chemotherapy [3,4]. Furthermore, PTEN affects radiosensitivity and is potential targets in glioblastoma, esophageal cancer, and nasopharyngeal cancer [35,36,37]. Epigenetic alterations can cause PTEN silencing through hypermethylation or mutations in the PTEN promoter region [8,9,10,11]. DNA methylation is associated with the transcriptional silencing of many genes in normal and malignant cells; such methylation is a key regulator of gene expression. The degree of methylation at CpG sites in 5-regulatory regions is crucial for the transcriptional activation of various genes in human cancers. PTEN gene expression is under the control of the CpG site methylation in its promoter region [7,13]. Based on previous data, a link between EPHA3 and PTEN expression was investigated in terms of epigenetic alteration. We found that EPHA3, DNMT1 and EZH2 are overexpressed with PTEN suppression in radioresistant head and neck cancer cells. We identified DNMT1 increase with EPHA3 overexpression as essential for PTEN gene promoter methylation and the subsequent decrease in PTEN expression in radiation resistance. Moreover, EPHA3 expression can regulate PTEN via EZH2 and H3K27me3 levels. We found that PTEN is suppressed strongly and consistently by the dual epigenetic regulation of DNA methylation and histone methylation. 

Epigenetic mechanisms such as DNA methylation, histone modification, chromatin remodeling, and non-coding RNAs have a key role in the development of the radiation resistance of cancer cells, as seen in our results. Many epigenetic drugs can revert the radioresistant phenotypes, and clinical trials are currently ongoing in epi-drugs combined with radiotherapy [38,39]. Epigenetic factors have an equally important role in drug resistance as radiation resistance [40,41,42,43]. The promoter hypermethylation of some genes was identified in cisplatin resistant ovarian cancer cells [40]. DNA methyltransferase inhibitor led to re-expression of genes shown to be preferentially methylated and silenced at relapse of pediatric acute lymphoblastic leukemia [41] and induced reverse chemoresistance in heterogeneous multiple myeloma [43]. Recent study showed that epigenetic therapy targeting EZH2 and DNMT1 could be a potential novel strategy for augmenting immunotherapy for hepatocellular carcinoma [44]. 

This study proved that C-myc might mediate a functional link between EPHA3 and DNMT1/EZH2, which regulates PTEN/Akt-based radiation resistance. In previous reports, combined MYC activation and PTEN loss induced genomic instability and aggressive prostate cancer [45], and C-myc modulated genes associated with oncogenesis in glioblastoma multiformes cells through deregulation of miRNAs via the c-Myc-miR-26a-PTEN signaling pathway [23]. However, the role of EPHA3 and C-myc, and their relationship with the PETN/Akt pathway in radioresistance, remains unclear. We demonstrated that EphA3 signaling induces the epigenetic silencing of PTEN by DNMT and EZH2, and that c-Myc is required in this process.

**Figure 5:**
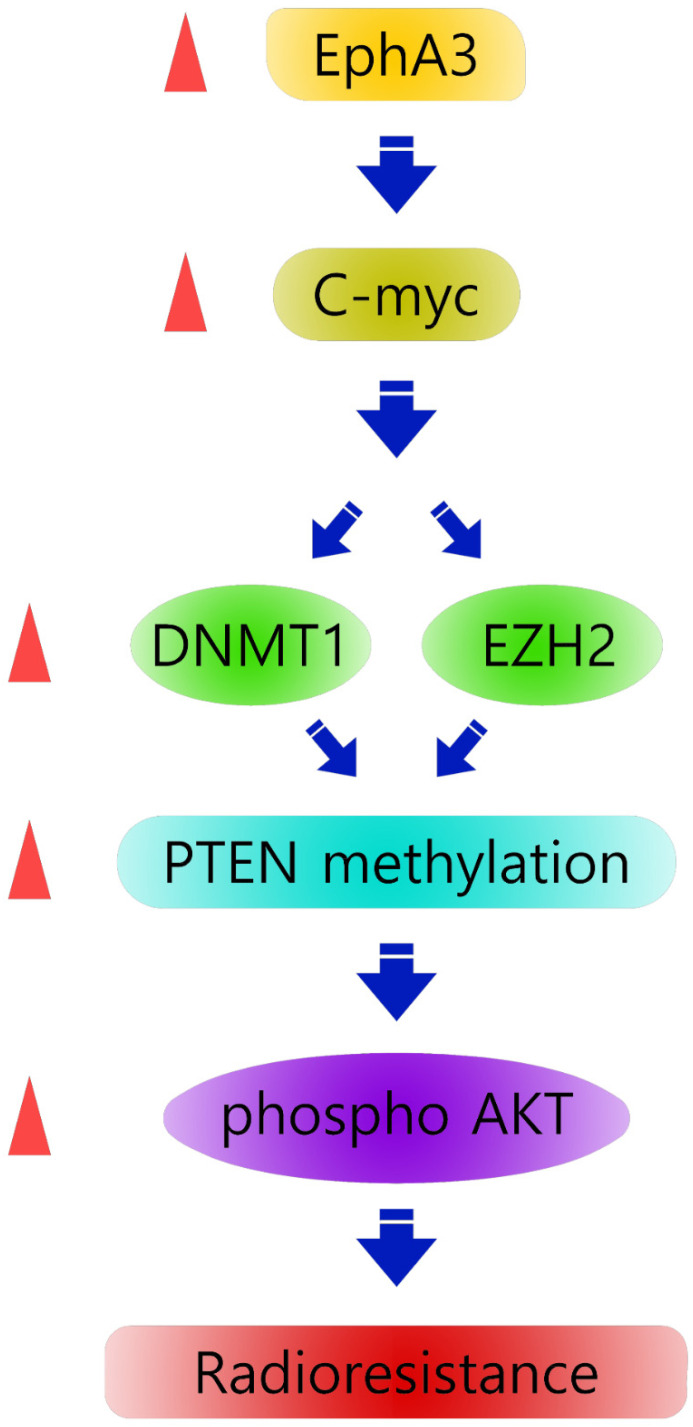
Our hypothetical model shows the downregulation of PTEN expression in radiation-resistant cells. PTEN expression is downregulated by CpG hypermethylation in the PTEN promoter (DNMT1-mediated DNA methylation) and histone hypermethylation in H3K27me3 (EZH2-mediated histone methylation), with constitutive activation of the Akt pathway. EPHA3/c-Myc could regulate PTEN expression epigenetically by dual mechanisms.

Our findings suggest a model (Figure 5) in which EPHA3 signaling induces the epigenetic silencing of PTEN through both the upregulation of DNMT1-mediated DNA methylation and EZH2-mediated histone methylation via c-Myc. Thus, the present study demonstrates a previously unrecognized epigenetic mechanism of PTEN regulation in radioresistant head and neck cancer cells. 

Previously, we established a radioresistant cell line with response to radiation and used DNA microarray data for identification of gene expression. Although genomic instability is predictable in radioresistant cell lines, a full genomic analysis was not performed. Further exploration of the results of the present study may help in the complete realization of the potential role of EPHA3 and PTEN in radioresistance.

## 5. Conclusions

Our results indicate that EPHA3 is a novel biomarker essential to the development of radioresistance. This protein induces PTEN suppression through epigenetic regulation, specifically, DNMT1- and EZH2-mediated hypermethylation. A greater understanding of the resistance mechanisms related to EPHA3 and PTEN in this study will enable the rational design of combination regimens and sequential treatment algorithms to improve clinical outcomes. Further studies will help to fully clarify the role of EPHA3 in radioresistant recurrent cancer treatment.

## Figures and Tables

**Table 1:** EPHA3, C-myc, and PTEN expression in laryngeal cancer specimens in tissue microarrays. (N = 104 specimens).

	Primary Surgery Specimens (N = 59)	Recurred Cancer Specimens (N = 45)	*p* Value
EPHA3			
Negative	28	11	0.016
Positive	31	34	
c-Myc			
Negative	30	23	0.040
Positive	22	29	
PTEN			
Negative	23	30	0.006
Positive	32	19	

## Data Availability

The data that support the findings of this study are available from the corresponding author upon reasonable request.

## Supplementary Materials

The following are available online at https://www.mdpi.com/article/10.3390/biom11040599/s1, Figure S1: EPHA3 expression was confirmed after EPHA3 transfection and silencing after 3 and 7 days; Figure S2: EPHA3, c-Myc and PTEN expression was evaluated in laryngeal cancer specimen using tissue microarray (X200); Figure S3: PTEN is suppressed with elevated EPHA3, c-Myc, DNMT1, and EZH2 in radioresistant cell lines. (A) Expression of EPHA3, c-Myc, DNMT1, EZH2 and PTEN in HN31R cell line and MDA-MB-231R cell line, comparing with the parent cell line on western blot; Table S1: Clinicopathological variables of samples in tissue microarray.
