# Supplementary material for: EPHA3 Contributes to Epigenetic Suppression of PTEN in Radioresistant Head and Neck Cancer
Source: Biomolecules. 2021 Apr 18;11(4):599. doi: 10.3390/biom11040599 (PMC8073943; doi:10.3390/biom11040599)
Supplement: Supplementary file 1 [file biomolecules-11-00599-s001.zip › Supplementary Table 1.pdf]

**Supplementary Table 1. Clinicopathological variables of samples in tissue microarray**

| | Primary surgery specimen (N=59) | Recurred cancer specimen (N=45) |
|---|---|---|
| Male:Female | 54:5 | 43:2 |
| Median age (y) | 69 | 68 |
| Tumor site | | |
| Supraglottis | 27 | 10 |
| Glottis | 20 | 27 |
| Subglottis | 2 | 3 |
| Transglottis | 10 | 5 |
| Tumor stage | | |
| I | 0 | 5 |
| II | 0 | 8 |
| III | 18 | 20 |
| IV | 41 | 12 |
