# Supplementary figures and images for: EPHA3 Contributes to Epigenetic Suppression of PTEN in Radioresistant Head and Neck Cancer
Source: Biomolecules. 2021 Apr 18;11(4):599. doi: 10.3390/biom11040599 (PMC8073943; doi:10.3390/biom11040599)

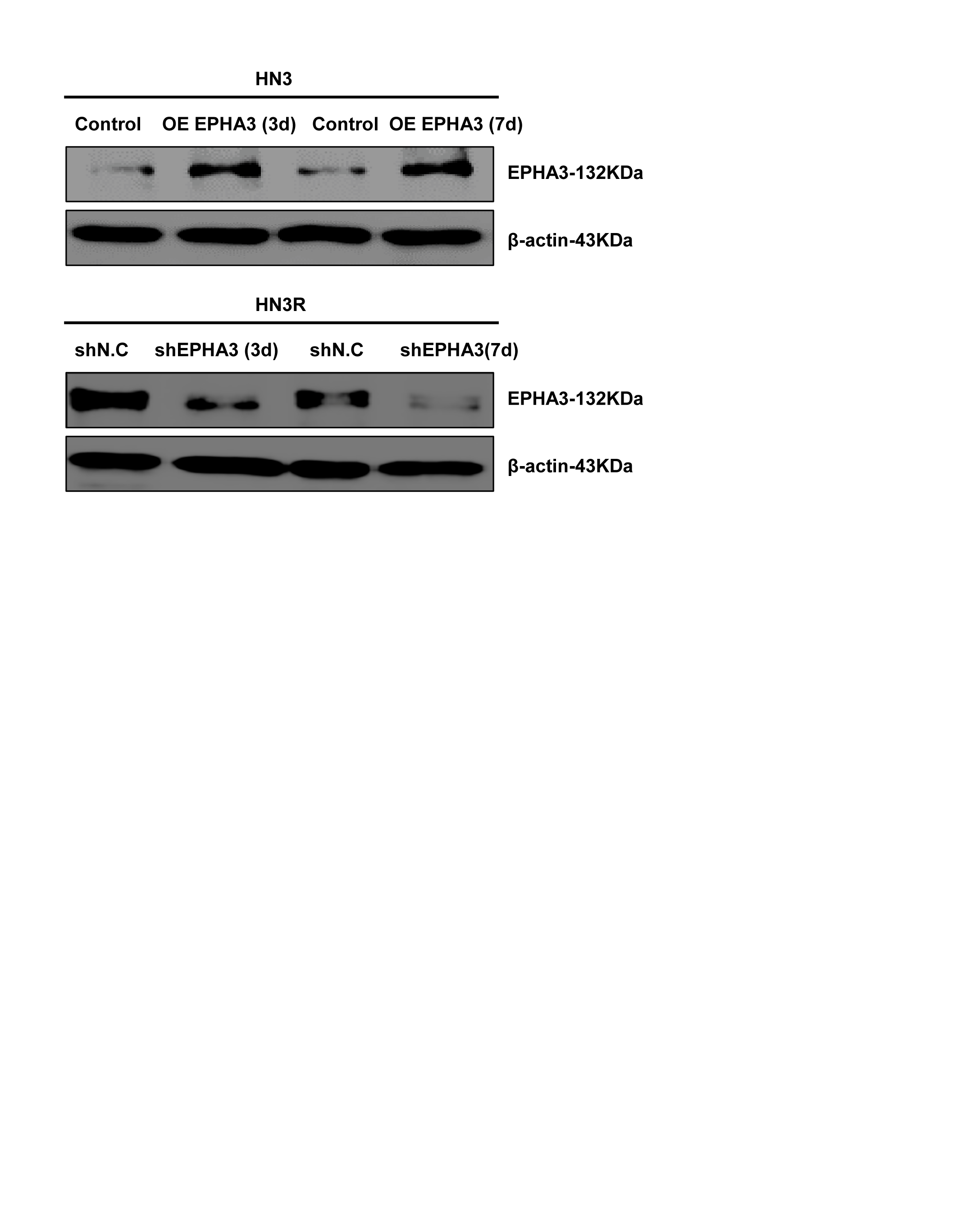

Supplement: Supplementary file 1 [file biomolecules-11-00599-s001.zip › Supplementary figure 1.tif]

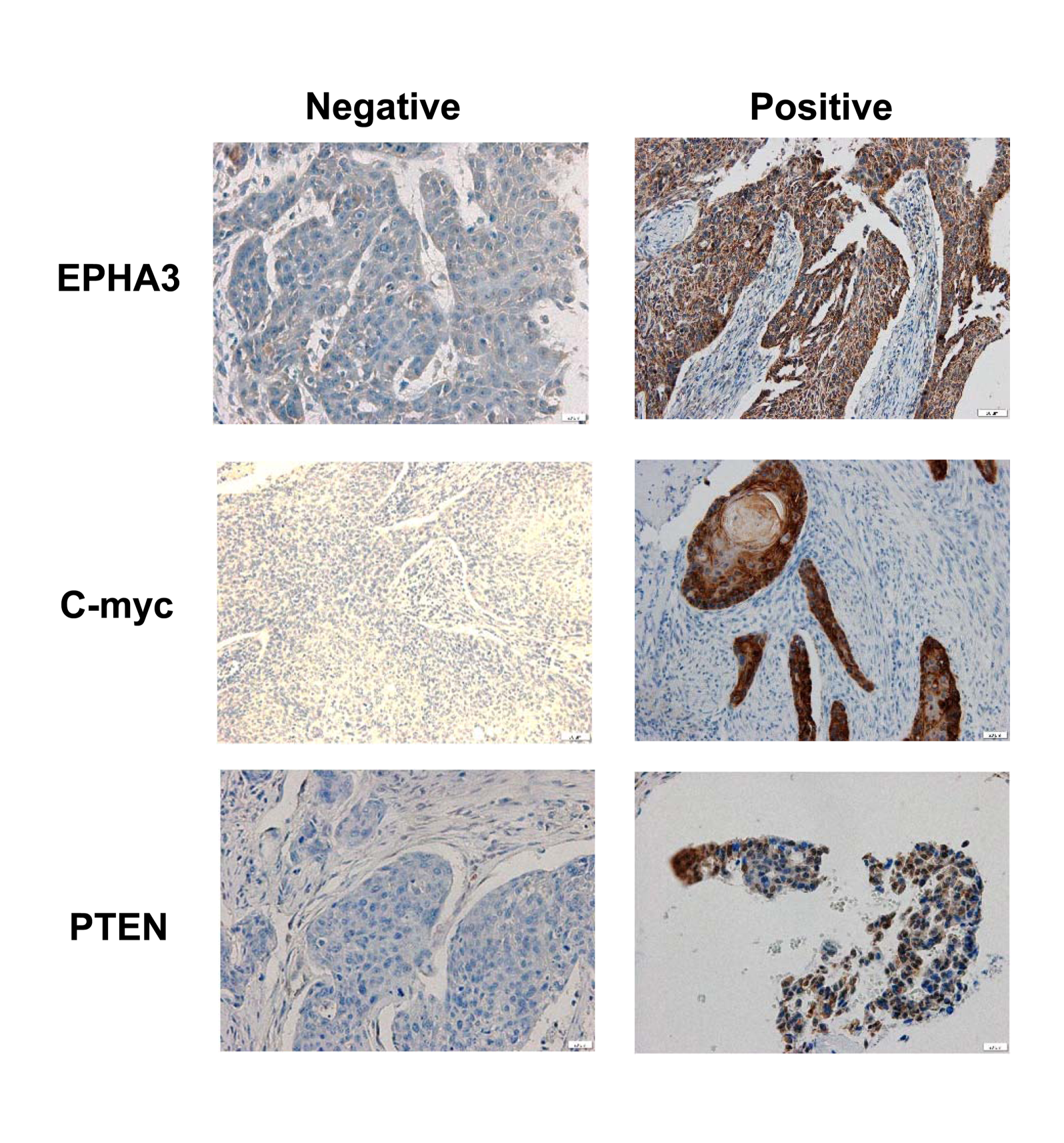

Supplement: Supplementary file 1 [file biomolecules-11-00599-s001.zip › Supplementary figure 2.tif]

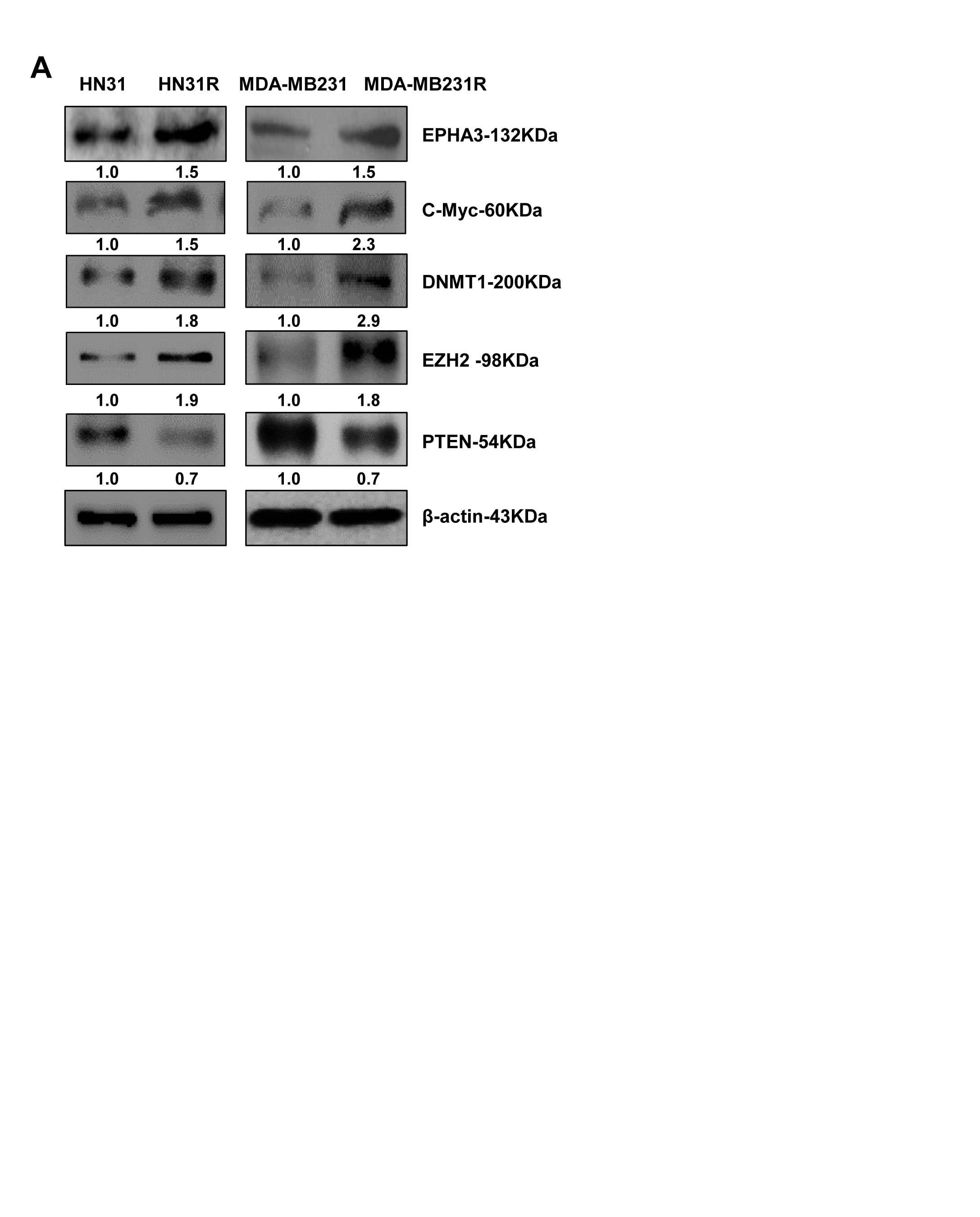

Supplement: Supplementary file 1 [file biomolecules-11-00599-s001.zip › Supplementary figure 3 .tif]
